# Acquired Haemophilia A Associated With Suspected Chronic Neutrophilic Leukaemia Presenting As Fatal Retroperitoneal Haemorrhage in an Elderly Patient

**DOI:** 10.7759/cureus.87400

**Published:** 2025-07-06

**Authors:** Fahad Ul Islam Mir, Usama Ali Nizam, Azhar H Baba, Sumiya Arshad, Saalis Maqbool, Mehreen Mir

**Affiliations:** 1 Acute Medicine, University Hospitals Bristol and Weston NHS Foundation Trust, Weston-super-Mare, GBR; 2 Endocrinology and Diabetes, University Hospitals Bristol and Weston NHS Foundation Trust, Weston-super-Mare, GBR; 3 Radiology, Islamabad Diagnostic Centre, Lahore, PAK; 4 General Internal Medicine, Government Medical College, Srinagar, Srinagar, IND; 5 Internal Medicine, International Medical College, Tongi, BGD

**Keywords:** acquired hemophilia a, chronic neutrophilic leukemia, factor viii inhibitor, prolonged aptt, retroperitoneal haemorrhage

## Abstract

Acquired haemophilia A (AHA) is a rare but potentially life-threatening bleeding disorder caused by autoantibodies against coagulation factor VIII. It often presents with spontaneous bleeding and prolonged activated partial thromboplastin time (aPTT) and is commonly associated with autoimmune disorders, malignancy, or idiopathic causes. Chronic neutrophilic leukaemia (CNL) is a rare myeloproliferative neoplasm that has occasionally been linked with paraneoplastic phenomena, including AHA.

An elderly patient presented following a fall with normal imaging but was noted to have a markedly prolonged aPTT (78-104 seconds), normal prothrombin time (PT), and an isolated severe factor VIII deficiency (<0.01 IU/mL). A Bethesda assay confirmed high-titer factor VIII inhibitors, with a measured level of 9 Bethesda units (BU). No underlying autoimmune disease or malignancy was identified initially; however, persistent neutrophilia over several years raised suspicion of undiagnosed CNL. The patient subsequently developed retroperitoneal haemorrhage with a significant haemoglobin drop, requiring transfusion. Later, the patient suffered an ST-elevation myocardial infarction (STEMI) and, after a multidisciplinary discussion, was transitioned to palliative care. Sadly, the patient passed away eight days post-admission.

This case highlights the diagnostic and therapeutic challenges of AHA, particularly when associated with occult or undiagnosed haematological malignancies like CNL. Early recognition and immunosuppressive therapy are crucial to improving outcomes.

## Introduction

Acquired haemophilia A (AHA) is a very uncommon autoimmune bleeding disorder resulting from the development of neutralizing antibodies (inhibitors) against coagulation factor VIII. The incidence is approximately 1-1.5 cases per million per year, predominantly affecting the elderly, and carries high morbidity and mortality due to severe bleeding events [1,2]. Clinical presentation may include spontaneous bleeding, soft tissue hematomas, and mucosal or retroperitoneal haemorrhages, often without prior bleeding history.

Risk factors for AHA include autoimmune conditions, malignancies, dermatological diseases, pregnancy, and medications, though up to 50% of cases are idiopathic [3]. Laboratory findings typically show an isolated prolongation of activated partial thromboplastin time (aPTT) not corrected by mixing studies, with reduced factor VIII levels and a positive Bethesda inhibitor assay.

Chronic neutrophilic leukaemia (CNL) is a rare myeloproliferative neoplasm characterized by sustained mature neutrophilia and splenomegaly. Its association with paraneoplastic syndromes, including acquired haemophilia, has rarely been reported [4]. This case underscores a possible link between undiagnosed CNL and AHA, with fatal bleeding complications.

## Case presentation

An elderly patient was admitted following a fall. CT trauma series including CT head was all negative. Initial blood investigations (Table 1) revealed leukocytosis, with a white cell count (WCC) of 13.4 ×10^9^/L, absolute neutrophil count (ANC) of 11.0 ×10^9^/L, a prolonged aPTT of 78 seconds (reference: 28-40 seconds), a normal prothrombin time (PT), and a normal C-reactive protein (CRP). A review of history, medications, and prior coagulation profiles (normal PT and aPTT in August 2024) revealed no obvious aetiology. A repeat aPTT was 83 seconds.

**Table 1:** Admission blood test results. MCV: mean corpuscular volume; CRP: C-reactive protein; aPTT: activated partial thromboplastin time; PT: prothrombin time; INR: international normalized ratio; ALT: alanine transaminase

Blood test	Result	Reference range
White cell count	13.4	4-11 x 10^9^/L
Haemoglobin	88	130-170 g/L
MCV	94	83-100 fL
Platelet count	239	150-400 x 10^9^/L
Neutrophil count	10.78	1.5-8.0 x 10^9^/L
CRP	2	<5 mg/L
aPTT	78	25.1-36.5 seconds
PT	11.7	10-13.2 seconds
INR	1.0	0.9-1.3
Fibrinogen	3.6	2-4 g/L
Creatinine	144	59-104 umol/L
Urea	16.6	2.5-7.8 mmol/L
Bilirubin	10	<21 umol/L
ALT	16	10-60 U/L

**Figure 1:**
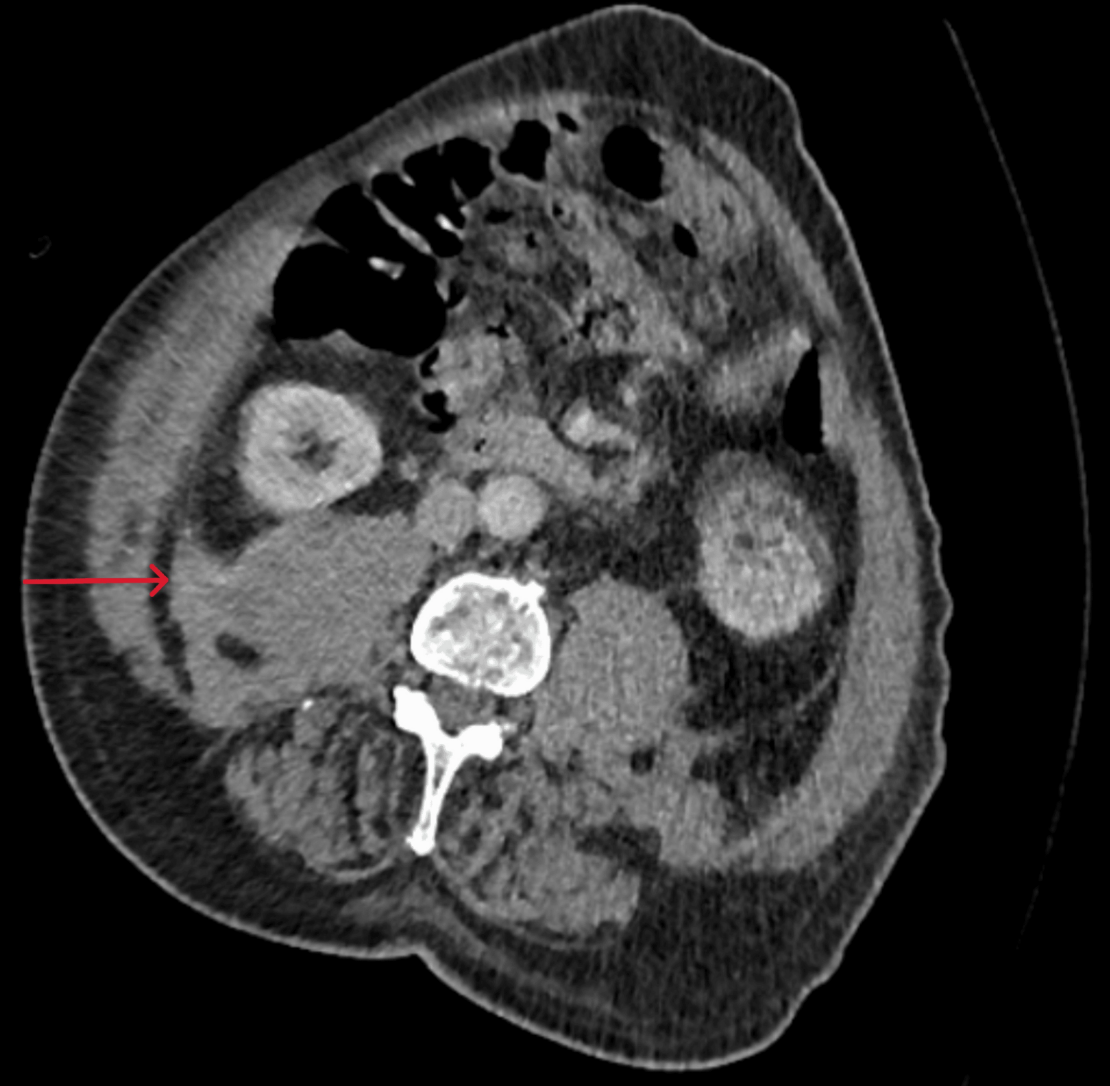
CT abdomen showing high-density fluid along the right psoas muscle, suggestive of hematoma (red arrow).

On day 1 of admission, the patient developed a spontaneous right arm bruise. Later that day, the patient became hypotensive and reported bilateral flank pain, along with a new right flank cutaneous hematoma. Haemoglobin dropped acutely from 88 g/L to 57 g/L, prompting a major haemorrhage call. Two units of packed red blood cells were transfused. CT abdomen/pelvis revealed a retroperitoneal haemorrhage with psoas muscle hematoma, without active extravasation (Figure 1). CT abdomen additionally showed hepatosplenomegaly, which was noted on previous scans as well. His BP and hemodynamic status improved. Sometime later, the patient reported central chest discomfort and was visibly sweaty. ECG was performed, which showed transient ST-elevation in inferior leads. Cardiology advice was sought who advised against treatment for chest pain and ECG changes and suggested to treat bleeding tendency. Interestingly, his chest pain improved after some time, along with the resolution of ST-segment changes. The case was discussed with the haematology team regarding suspicion of acquired haemophilia, who suggested repeating the coagulation screen and sending for a haemophilia screen (Table 2). WCC was raised, and the ANC was 12.64, representing approximately 88% of the total cells. Blood film showed neutrophilia, and there were no blast cells were seen. The repeat aPTT was 104 seconds. Mixing studies showed partial correction (to 73.3 sec), and the Bethesda assay confirmed FVIII inhibitors (9 Bethesda Units). The autoimmune screen was negative apart from weakly positive antinuclear antibody (ANA), with negative extractable nuclear antigen (ENA) and Hep2 ANA. Myeloma screen and lupus anticoagulant were negative. Factor assays showed factor VIII <0.01 IU/mL (reference: 0.5-1.5 IU/mL), with normal levels of factors IX, XI, and XII, and von Willebrand factor (vWF) activity was elevated (3.19 IU/mL; reference: 0.40-1.63).

**Table 2:** Repeat blood test results following the development of flank hematoma and evaluation of prolonged aPTT. INR: international normalized ratio; CRP: C-reactive protein; ANA: antinuclear antibody; ENA: extractable nuclear antigen

Blood test	Results	Reference range
White cell count	14.6	4-11 x 10^9^/L
Haemoglobin	57	130-170 g/L
Platelet count	308	150-400 x 10^9^/L
Neutrophil count	12.64	1.5-8 x 10^9^/L
Activated partial thromboplastin time (aPTT)	104	25.1-36.5 seconds
Prothrombin time (PT)	12.8	10-13.2 seconds
INR	1.1	1.0-1.3
Fibrinogen	>6	2-4 g/L
CRP	46	<5 mg/L
ANA	Weakly positive	-
Hep2 ANA	Negative	-
ENA	Negative	-
Anti-dsDNA	23.7	<27 IU/mL
Rheumatoid factor	13.3	<20 IU/mL
Serum kappa: lambda ratio	1.14	0.26-1.25
Factor VIII assay	0.01	0.50-2.00 IU/mL
Factor XI assay	0.74	0.7-2.00 IU/mL
Factor XII assay	0.50	0.5-2.00 IU/mL
Factor IX assay	0.73	0.5-2.00 IU/mL
von Willebrand activity	3.19	0.4-1.63 IU/mL
Immunoglobulin G	11.66	6-16 g/L
Immunoglobulin M	0.50	0.4-2.3 g/L
Immunoglobulin A	2.97	0.7-4.0 g/L
Mixing studies 50:50	73.3 seconds	-
Bethesda assay	9 Bethesda units	-

Historical full blood counts revealed persistent neutrophilia over a three-year period, with the most recent results in August 2024 showing a WCC of 14 ×10^9^/L and an ANC of 11.2 ×10^9^/L, raising suspicion of previously undiagnosed CNL. No lesions were seen on CT to suggest solid malignancy. Treatment was complicated by the fact that he was already bleeding, plus he had an ischemic cardiac event, and after a multidisciplinary discussion, he was switched to palliative care. The patient sadly passed away eight days post-admission.

## Discussion

This case illustrates a classic presentation of AHA with spontaneous bleeding and isolated prolonged aPTT in an elderly patient, with no prior history of bleeding disorders. The diagnosis was supported by low FVIII levels, incomplete correction on mixing studies, and positive Bethesda assay.

The absence of a clear autoimmune or solid tumour aetiology led to a haematological review, with longstanding neutrophilia raising the suspicion of CNL, a rare myeloproliferative neoplasm. Although not confirmed by bone marrow biopsy due to the rapid clinical deterioration, this represents a likely paraneoplastic-acquired haemophilia. AHA can present as life-threatening bleeds including retroperitoneal haemorrhage, as seen in this case, often requiring urgent immunosuppressive therapy, bypassing agents, and specialist care [5,6]. Unfortunately, rapid progression and cardiac complications limited definitive intervention in this patient.

Early recognition and prompt initiation of factor VIII inhibitor bypassing therapy (e.g., recombinant activated factor VII (rFVIIa), activated prothrombin complex concentrate (FEIBA)) and immunosuppressive treatment (e.g., corticosteroids, cyclophosphamide, rituximab) are critical in reducing mortality in AHA [3,6].

## Conclusions

This case highlights the diagnostic complexity and clinical severity of AHA, particularly when associated with a potential underlying myeloproliferative disorder such as CNL. The patient’s presentation with spontaneous bleeding, markedly prolonged aPTT, and severe factor VIII deficiency in the absence of prior coagulopathy emphasizes the importance of considering acquired haemophilia in the differential diagnosis of unexplained bleeding. Early recognition of this rare condition, with prompt initiation of appropriate treatment with immunosuppressive therapy and haemostatic agents, is essential to improving outcomes. Unfortunately, the delayed diagnosis in this case, compounded by retroperitoneal haemorrhage and subsequent cardiovascular complications, underscores the high mortality risk associated with AHA and the need for heightened clinical awareness, particularly in elderly patients with unexplained bleeding and abnormal coagulation profiles.

## References

[1] Collins PW, Hirsch S, Baglin TP (2007). Acquired hemophilia A in the United Kingdom: a 2-year national surveillance study by the United Kingdom Haemophilia Centre Doctors' Organisation. Blood.

[2] Knoebl P, Marco P, Baudo F (2012). Demographic and clinical data in acquired hemophilia A: results from the European Acquired Haemophilia Registry (EACH2). J Thromb Haemost.

[3] Franchini M, Targher G, Manzato F, Lippi G (2008). Acquired factor VIII inhibitors in oncohematology: a systematic review. Crit Rev Oncol Hematol.

[4] Szuber N, Elliott M, Tefferi A (2020). Chronic neutrophilic leukemia: 2020 update on diagnosis, molecular genetics, prognosis, and management. Am J Hematol.

[5] Tiede A, Collins P, Knoebl P (2020). International recommendations on the diagnosis and treatment of acquired hemophilia A. Haematologica.

[6] Kruse-Jarres R, St-Louis J, Greist A (2015). Efficacy and safety of OBI-1, an antihaemophilic factor VIII (recombinant), porcine sequence, in subjects with acquired haemophilia A. Haemophilia.

